# Inverse association between triglyceride glucose index and muscle mass in Korean adults: 2008–2011 KNHANES

**DOI:** 10.1186/s12944-020-01414-4

**Published:** 2020-11-22

**Authors:** Sung-Ho Ahn, Jun-Hyuk Lee, Ji-Won Lee

**Affiliations:** 1 Department of Family Medicine, Yonsei University College of Medicine, Seoul, Republic of Korea; 2 Department of Family Medicine, Yonsei University College of Medicine, Gangnam Severance Hospital, 211 Eonju-ro, Gangnam-gu, Seoul, 06273 Republic of Korea; 3 Department of Family Medicine, Yonsei University College of Medicine, Yongin Severance Hospital, 363, Dongbaekjukjeon-daero, Giheung-gu, Yongin-si, Gyeonggi-do 16995 Republic of Korea

**Keywords:** Triglyceride and glucose index, Sarcopenia, Low skeletal muscle index, Public health, Insulin resistance, Chronic inflammation, Inflammatory cytokines, Metabolic syndrome

## Abstract

**Background:**

Since sarcopenia is an important risk factor for falls or cardiovascular disease, early detection and prevention of sarcopenia are being increasingly emphasized. Emerging evidence has indicated relationships between sarcopenia, insulin resistance, and inflammation. The triglyceride glucose (TyG) index, a novel surrogate marker of insulin resistance and systemic inflammation, has not yet been shown to be associated with sarcopenia. This study aimed to examine the relationship between the TyG index and muscle mass in Korean adults.

**Methods:**

This study included 15,741 non-diabetic adults over 19 years old using data from the 2008–2011 Korea National Health and Nutrition Examination Survey. Participants were divided into three groups according to tertiles of the TyG index. A low skeletal muscle mass index (LSMI) was defined by the Foundation for the National Institutes of Health Sarcopenia Project criteria. A weighted multivariate logistic regression model was used to analyze relationships between TyG index tertiles and LSMI.

**Results:**

The ORs (95% CIs) for LSMI in the second and third TyG tertiles, compared to the first tertile, were 1.463 (1.131–1.892) and 1.816 (1.394–2.366), respectively, after adjusting for confounding factors. Higher TyG index values were also associated with increased odds of LSMI in adults under 65 years who did not exercise regularly, who consumed less than 30 g of alcohol per day, who did not currently smoke, and who ate less than 1.5 g of protein/kg/day.

**Conclusion:**

The TyG index was significantly and positively associated with LSMI in Korean adults.

**Supplementary Information:**

The online version contains supplementary material available at 10.1186/s12944-020-01414-4.

## Introduction

Sarcopenia is defined as an unintentional decline in muscle mass, strength, and performance that arises with aging [1]. However, there are no unified criteria for diagnosing sarcopenia [2–5]. Typically, muscle mass reduces by 3–8% per decade after the age of 30, and muscle strength [6, 7], a major part of physical function, decreases by 1–2% per year after 50 years of age [8, 9]. For this reason, sarcopenia is mainly observed in older adults, although it can also occur in younger adults [10]. The estimated prevalence of sarcopenia has been reported to be at least 4.6% in all older adults and nearly 25% in older hospitalized patients [11]. Sarcopenia has become an increasingly important issue in public health as life spans have increased [12, 13]. Indeed, healthcare costs related to sarcopenia were reported to be $18.5 billion ($10.8 billion in men, $7.7 billion in women) in the United States in 2000, constituting about 1.5% of total healthcare expenditures for the year [13, 14].

Sarcopenia is associated not only to increased risks of falls, fractures, and disabilities [15, 16], but also to metabolic disorders, such as insulin resistance, type 2 diabetes mellitus, dyslipidemia, and hypertension [17], as a result of decreases in basal metabolic rate, fat-free mass, and physical activity. Additionally, sarcopenia has been shown to be associated with chronic, low-grade, systemic inflammation [18–21], and several reports have described a relationship between sarcopenia, insulin resistance [22], and inflammation [23].

The triglyceride and glucose index (TyG index), a product of triglyceride levels and fasting plasma glucose concentration, has recently been recognized as a useful clinical surrogate marker of insulin resistance [24, 25] and systemic inflammation [26, 27]. Moreover, the TyG index has been validated for predicting the risk of diabetes and cardiovascular disease syndrome [28–32], as well as for diagnosing metabolic syndrome [33]. However, to date there have been no studies of the relationship between the TyG index and sarcopenia. In this analysis, we investigated the association between TyG index and muscle mass in a representative sample of Korean adults.

## Materials and methods

### Study population

All study data were obtained from the 2008–2011 Korea National Health and Nutrition Examination Survey (KNHANES), a nationwide representative survey conducted by the Korea Centers for Disease Control and Prevention to assess the health and nutritional status of Koreans. The KNHANES employs a cross-sectional, stratified, multistage, probability sampling design based on age, sex, and geographical area. Sample weights were assigned to subjects to capture a sample representing the general Korean population. Survey items in the KNHANES have been revised partially because of the availability of survey resources, and some questionnaires have been updated and changed over the years [34]. Detailed methods related to the KNHANES have been previously described [34].

**Fig. 1:**
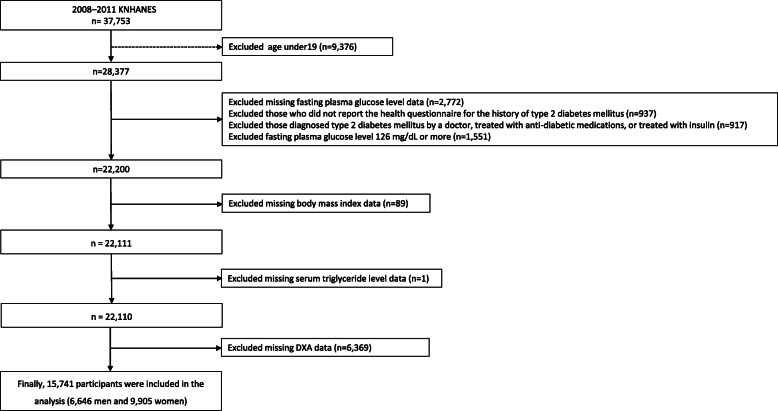
Flowchart of study population selection

A total 37,753 of individuals participated in the 2008–2011 KNHANES. Of these participants, we excluded 1) those who were under 19 years old (*n* = 9736), 2) those missing serum glucose level data (*n* = 2772), 3) those who did not report history of type 2 diabetes mellitus (*n* = 937), 4) those diagnosed type 2 diabetes mellitus by a doctor, treated with anti-diabetic medications, or treated with insulin (*n* = 917), 5) those who had fasting plasma glucose levels 126 mg/dL or more (*n* = 1551), 6) those missing body mass index (BMI) data (*n* = 89), 7) those missing serum triglyceride level data (n = 1), or 8) those missing whole-body dual-energy X-ray absorptiometry (DXA) data (*n* = 6369). Finally, a total of 15,741 participants (6646 men and 9095 women) were included in this analysis (Fig. 1).

### Biochemical measurements

Blood samples were collected from the antecubital vein from each patient after at least 8 h of fasting. Serum total cholesterol, triglycerides, high-density lipoprotein (HDL) cholesterol, and plasma glucose concentrations were measured using a Hitachi 7600 Analyzer. White blood cell (WBC) counts were analyzed using a XE-2100D blood cell counter. The TyG index was calculated as follows: log [serum triglycerides (mg/dL) × plasma glucose (mg/dL)/2]. At present, there is no reference value for the prediction of sarcopenia using the TyG index. Therefore, we divided participants into three groups according to tertiles of the TyG index: T1 (6.45–8.19), T2 (8.20–8.71), and T3 (8.72–11.29).

### Assessment of muscle mass

DXA was performed from July 2008 to June 2011 to evaluate body composition (QDR 4500A; Hologic Inc., Bedford, MA, USA). Body composition data were obtained from predefined anatomical areas as follows: head, arms, legs, trunk, pelvic region, and whole body. Participants were analyzed for bone mineral content (g), bone mineral density (g/cm^2^), fat mass (g), lean body mass (g), and total fat percentage (fat mass/total mass × 100). Skeletal muscle mass was calculated using the following equation: lean body mass (g) - bone mineral content (g). Appendicular skeletal muscle mass (ASM) was calculated as the summation of the skeletal muscle mass of both upper and lower extremities. We defined the skeletal muscle mass index (SMI) as ASM (kg) divided by body mass index (BMI) (kg/m^2^). Finally, the low skeletal muscle index (LSMI) was defined according to the Foundation for the National Institutes of Health (FNIH) Sarcopenia Project criteria: SMI values less than 0.789 for men and less than 0.512 for women [5].

### Measurement of anthropometric and clinical parameters

Height (cm) and body weight (kg) were estimated to the nearest 0.1 cm using a stadiometer without shoes in a standing posture or supine position and to the nearest 0.1 kg using digital scale in light clothing, respectively. BMI was calculated as body weight divided by height squared (kg/m^2^). Adults with a BMI greater than or equal to 25 kg/m^2^ were considered overweight and those with a BMI less than 18.5 kg/m^2^ were considered underweight according to the guidelines of the International Obesity Task Force of the World Health Organization [35]. Adults were categorized into three different categories of smoking status: current smokers, ex-smokers, and never smokers. We defined a current smoker as someone who smoked at the time of the interview and had smoked at least 100 cigarettes over their lifetime. Ex-smokers were defined as those who did not currently smoke, but who had smoked at least 100 cigarettes in his or her lifetime. Heavy alcohol use was defined as consuming an average of 30 g or more of alcohol per day. We defined regular exercise as 20 min of vigorous exercise at least 3 days per week or 30 min of moderate exercise/walking at least 5 days per week, and physical activity was assessed using the International Physical Activity Questionnaire (IPAQ). A food frequency questionnaire (FFQ) was used for all adults aged 19 years or older. Daily nutritional intake of total calorie (kcal/day), carbohydrate (CHO) (g/day), fat (g/day), and protein (g/day) were surveyed. Based on the modified NCEP ATP-III criteria [36], this study defined metabolic syndrome according to the following criteria: (1) waist circumference ≥ 90 cm in men and ≥ 85 cm in women, per the Korean-specific cut-offs for abdominal obesity of the Korean Society of Obesity [37]; (2) serum triglycerides ≥150 mg/dL; (3) either fasting plasma glucose ≥100 mg/dL, the use of anti-diabetic medications, or current treatment with insulin therapy; (4) HDL cholesterol < 40 mg/dL in men or < 50 mg/dL in women or use of lipid-lowering medications; and (5) blood pressure ≥ 130/85 mmHg or use of anti-hypertensive medications. Detailed information about the KNHANES is available on the KNHANES website (http://knhanes.cdc.go.kr).

### Statistical analysis

All data are presented as a mean or percentage (%) ± standard error (SE). For the analysis of clinical characteristics of the study population, a weighted analysis of variance (ANOVA) test was used for continuous variables, followed by post-hoc analysis with Bonferroni correction. For categorical variables, weighted chi-square tests were used to analyze differences among the three groups, followed by post-hoc analysis with Bonferroni correction. After adjusting for confounding variables, a weighted multivariate logistic regression analysis was performed to calculate odds ratios (ORs) with 95% confidence intervals (CIs) for LSMI according to the TyG index tertiles. We further analyzed subgroups according to age, alcohol drinking status, smoking status, amount of protein intake, and regular exercise through a weighted multivariate logistic regression analysis. All statistical analyses were conducted using SPSS statistical software (version 25.0; SPSS Inc., Chicago, IL, USA). The significance level was set at *p* less than 0.05.

## Results

### General characteristics of the study population

**Fig. 2:**
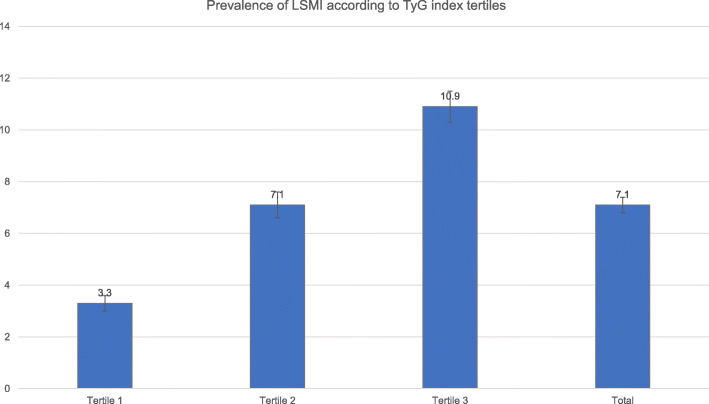
Prevalence of LSMI according to TyG index tertiles

Table 1 presents the clinical characteristics of 15,741 subjects according to tertiles of the TyG index. The proportions of men and mean age were lowest in T1 and highest in T3. The mean values of waist circumference, BMI, mean blood pressure, blood leukocyte count, fasting plasma glucose levels, serum total cholesterol levels, and log-transformed triglyceride levels increased, whereas HDL cholesterol levels decreased with each increase in TyG index tertile. The proportions of heavy alcohol drinkers, current smokers, and number of chronic diseases increased along with TyG index tertiles. The proportion of adults participating in regular exercise was highest in T1 and lowest in T3, although these trends were not statistically significant. The average amounts of daily calorie intake, carbohydrate intake, and protein intake significantly increased with increasing TyG index tertiles, while the amount of daily fat intake decreased with increasing TyG index tertiles. The mean values of SMI decreased with increasing TyG index tertiles for both men and women. The prevalence of LSMI increased with increasing TyG index tertiles (Fig. 2). The number of components of metabolic syndrome was higher in T3 than T1. WBC counts increased with an increase in the TyG index tertiles.

**Table 1:** Clinical characteristics of three different population

TyG index	2008–2011 KNHANES				
	T1	T2	T3	Total	*p* for trend
	6.45–8.19	8.20–8.71	8.72–11.29		
**Unweighted number**	5229	5235	5277	15,741	
**Male sex,n(%)**	1520 (29.1)	2193 (41.9)	2933 (55.6)	6641 (42.2)	< 0.001
**Age, years**	38.3 ± 0.3^a^	44.3 ± 0.3^b^	47.7 ± 0.3^c^	43.4 ± 0.2	< 0.001
**Waist circumference, cm**	75.2 ± 0.2^a^	80.2 ± 0.2^b^	85.5 ± 0.2^c^	80.3 ± 0.1	< 0.001
**Body weight, kg**	59.1 ± 0.2^a^	63.4 ± 0.2^b^	68.2 ± 0.2^c^	63.6 ± 0.1	< 0.001
**Mean blood pressure, mmHg**	85.4 ± 0.2^a^	90.3 ± 0.3^b^	94.5 ± 0.3^c^	90.1 ± 0.2	< 0.001
Leukocyte count (*1000/μL**)**	5.6 ± 0.0^a^	6.0 ± 0.0^b^	6.6 ± 0.0^c^	6.1 ± 0.0	< 0.001
**Glucose, mg/dL**	88.0 ± 0.1^a^	92.2 ± 0.2^b^	96.9 ± 0.2^c^	92.3 ± 0.1	< 0.001
**Log-transformed Triglyceride, mg/dL**	4.0 ± 0.0^a^	4.6 ± 0.0^b^	5.3 ± 0.0^c^	4.7 ± 0.0	< 0.001
**Employment status, % (SE)**	63.6 (0.9)^a^	62.9 (1.0)^a^	68.1 (0.9)^c^	64.8 (0.6)	< 0.001
**Heavy alcohol use, % (SE)**	5.2 (0.5)^a^	7.5 (0.5)^b^	13.8 (0.7)^c^	8.8 (0.3)	< 0.001
**Current smoker, % (SE)**	18.5 (0.8)^a^	25.3 (0.9)^b^	38.0 (1.0)^c^	27.0 (0.6)	< 0.001
**Regular exercise, % (SE)**	25.5 (0.8)^a^	25.4 (0.8)^a^	24.0 (0.8)^a^	25.0 (0.5)	0.307
**Daily calorie intake, kcal/day**	1982.3 ± 16.5^a^	2010.0 ± 17.3^a^	2125.7 ± 20.1^c^	2039.3 ± 11.1	< 0.001
**Daily protein intake, % of total calorie intake**	14.6 ± 0.1^a^	14.4 ± 0.1^a^	14.2 ± 0.1^b^	14.4 ± 0.1	0.001
**SMI**					
**Men**	1.006 ± 0.004^a^	0.963 ± 0.004^b^	0.921 ± 0.003^c^	0.963 ± 0.002	< 0.001
**Women**	0.666 ± 0.002^a^	0.629 ± 0.002^b^	0.596 ± 0.002^c^	0.631 ± 0.002	< 0.001
**Number of components of metabolic syndrome**					< 0.001
**0**	56.3 (0.9)^a^	30.9 (0.9)^b^	3.4 (0.4)^c^	30.6 (0.5)	
**1**	33.0 (0.8)^a^	39.5 (0.9)^b^	15.4 (0.7)^c^	29.3 (0.5)	
**2**	8.4 (0.5)^a^	20.0 (0.7)^b^	29.8 (0.9)^c^	19.2 (0.4)	
**3**	2.0 (0.2)^a^	8.3 (0.4)^b^	28.9 (0.9)^c^	12.9 (0.4)	
**4**	0.2 (0.1)^a^	1.3 (0.2)^b^	17.8 (0.6)^c^	6.3 (0.2)	
**5**	–	–	4.8 (0.3)	1.6 (0.1)	
**Number of chronic diseases, % (SE)**					< 0.001
**0**	95.6 (0.4)^a^	93.1 (0.4)^b^	92.1 (0.5)^b^	93.5 (0.3)	
**1**	4.0 (0.3)^a^	6.2 (0.4)^b^	7.2 (0.4)^b^	5.8 (0.2)	
**≥ 2**	0.4 (0.1)^a^	0.7 (0.1)^b^	0.7 (0.1)^b^	0.6 (0.1)	

*Abbreviations*: *TyG index* triglyceride-glucose index, *KNHANES* Korean National Health and Nutrition Examination Survey, *SE* standard error, *BMI* body mass index, *SMI* skeletal muscle mass index

*p* for trend was derived from weighted generalized linear regression analysis for continuous variables, followed by post-hoc analysis with Bonferroni correction and weighted chi-square test for linear-by-linear association for categorical variables, followed by post-hoc analysis with Bonferroni correction.

Superscripts (^a b c^), for a particular variable, indicate significant difference between tertiles (*p* < 0.0167). Variables with the same superscripts are not significantly different (*p* > 0.0167). When only one comparison was significant, one of the cell means has no superscript attached.

### Association between TyG index and LSMI

Table 2 shows the results of multivariate logistic regression analysis, including the ORs (95% CIs) for LSMI according to the TyG index tertiles. The OR (95% CIs) for LSMI in T3 of the TyG index versus T1 was 3.549 (2.897–4.347). This relationship remained significant after adjusting for age, sex, body weight, regular exercise, employment status, heavy alcohol use, smoking status, daily protein intake, number of chronic diseases, total cholesterol level, plasma glucose level, and mean blood pressure (T3 vs. T1, ORs = 1.816, 95% CIs: 1.394–2.366, *p* < 0.001).

**Table 2:** Association between TyG index and LSMI

TyG index tertile	Sample size of LSMI, n (%)	B	SE	ORs	95% CIs	Wald	*p*	Overall *p*	*p* for trend
Unadjusted						79.670		< 0.001	< 0.001
T1	228 (4.4)	Ref.	Ref.	1 (Ref.)	Ref.		Ref.		
T2	446 (8.5)	0.799	79.67	2.223	1.781–2.776		< 0.001		
T3	712 (13.5)	1.267	0.103	3.549	2.897–4.347		< 0.001		
Model 1						28.277		< 0.001	< 0.001
T1	228 (4.4)	Ref.	Ref.	1 (Ref.)	Ref.		Ref.		
T2	446 (8.5)	0.482	0.121	1.619	1.277–2.053		< 0.001		
T3	712 (13.5)	0.831	0.116	2.296	1.829–2.881		< 0.001		
Model 2						14.880		< 0.001	< 0.001
T1	228 (4.4)	Ref.	Ref.	1 (Ref.)	Ref.		Ref.		
T2	446 (8.5)	0.434	0.130	1.544	1.195–1.994		0.001		
T3	712 (13.5)	0.711	0.133	2.037	1.569–2.645		< 0.001		
Model 3						10.809		< 0.001	< 0.001
T1	228 (4.4)	Ref.	Ref.	1 (Ref.)	Ref.		Ref.		
T2	446 (8.5)	0.399	0.131	1.491	1.152–1.930		0.002		
T3	712 (13.5)	0.629	0.137	1.876	1.434–2.454		< 0.001		
Model 4						10.013		< 0.001	< 0.001
T1	228 (4.4)	Ref.	Ref.	1 (Ref.)	Ref.		Ref.		
T2	446 (8.5)	0.380	0.131	1.463	1.131–1.892		0.004		
T3	712 (13.5)	0.597	0.135	1.816	1.394–2.366		< 0.001		

*Abbreviations*: *TyG index* triglyceride-glucose index, *LSMI* low skeletal muscle mass index, *ORs* odds ratio, *CIs* confidence intervals

Model 1: Adjusted for age, sex, body weight, and regular exercise.

Model 2: Adjusted for variables included in Model 1 plus employment status, alcohol use, smoking status, daily protein intake, number of chronic diseases, and serum total cholesterol level.

Model 3: Adjusted for variables included in Model 2 plus plasma glucose level.

Model 4: Adjusted for variables included in Model 3 plus mean blood pressure./

SMI was defined using the following equation: ASM (kg)/BMI (kg/m2).

We defined the LSMI according to the cut-off value of SMI based on the Foundation for the National Institutes of Health (FNIH) sarcopenia project criteria: SMI less than 0.789 for men and SMI less than 0.512 for women.

The odds ratio and 95% confidence interval were calculated using the weighted multi-variate logistic regression analysis to evaluate the relationship between TyG index and LSMI.

Table 3 presents the results of subgroup analysis showing the relationship between TyG index and LSMI. The fully adjusted ORs (95% CIs) for LSMI in T3 compared to T1 were significant in both sex, adults under 65 years of age, both regular and irregular exercising groups, adults with normal body weight, drinking alcohol less than 30 g per day group, both current smokers and non-current smokers, both adults who consumed 1.5 g per kg of protein or more per day and adults who consumed less than 1.5 g per kg of protein per day. There were no significant differences between groups among adults over 65 years of age, underweight adults, overweight adults, and heavy alcohol drinkers.

**Table 3:** Subgroup analysis for relationships between TyG index tertiles and LSMI using multivariate logistic regression analysis

	T1	T2 ORs (95% CIs)	*p*	T3 ORs (95% CIs)	*p*	overall *p*	*p* for trend
Sex							
Men	1 (Ref.)	1. 485 (0.969–2.275)	0.069	2.138 (1.377–3.319)	0.001	0.001	< 0.001
Women	1 (Ref.)	1.415 (1.068–1.874)	0.016	1.620 (1.189–2.207)	0.002	0.010	0.003
Age groups							
< 65	1 (Ref.)	1.864 (1.363–2.549)	< 0.001	2.691 (1.947–3.719)	< 0.001	< 0.001	< 0.001
≥ 65	1 (Ref.)	1.177 (0.823–1.683)	0.371	1.333 (0.940–1.889)	0.107	0.248	0.095
Regular exercise							
Yes	1 (Ref.)	1.628 (0.925–2.866)	0.091	1.862 (1.074–3.229)	0.027	0.087	0.027
No	1 (Ref.)	1.400 (1.068–1.833)	0.015	1.795 (1.352–2.384)	< 0.001	< 0.001	< 0.001
Weight status							
Underweight	1 (Ref.)	1.201 (0.205–7.027)	0.839	0.085 (0.005–1.432)	0.087	0.228	0.132
Normal	1 (Ref.)	1.412 (1.018–1.959)	0.039	1.568 (1.085–2.266)	0.017	0.050	0.021
Overweight	1 (Ref.)	0.990 (0.685–1.430)	0.957	1.060 (0.747–1.505)	0.743	0.835	0.621
Heavy alcoholics							
< 30 g/day	1 (Ref.)	1.535 (1.206–1.954)	0.001	1.897 (1.470–2.448)	< 0.001	< 0.001	< 0.001
≥ 30 g/day	1 (Ref.)	0.334 (0.088–1.266)	0.107	0.668 (0.180–2.483)	0.547	0.133	0.997
Current smoker							
Yes	1 (Ref.)	1.167 (0.624–2.183)	0.629	2.097 (1.096–4.012)	0.025	0.009	0.008
No	1 (Ref.)	1.536 (1.190–1.983)	0.001	1.775 (1.363–2.313)	< 0.001	< 0.001	< 0.001
Protein intake							
> 1.5 g/kg/day	1 (Ref.)	1.133 (0.582–2.202)	0.713	2.160 (1.132–4.121)	0.020	0.008	0.010
≤ 1.5 g/kg/day	1 (Ref.)	1.515 (1.151–1.995)	0.003	1.816 (1.361–2.423)	< 0.001	< 0.001	< 0.001

*Abbreviations*: *TyG index* triglyceride-glucose index, *LSMI* low skeletal muscle mass index, *ORs* odds ratios, *CIs* confidence intervals

Each *P* was calculated by multivariate logistic regression analysis after adjusting for all confounders (age, sex, body weight, regular exercise, employment status, alcohol use, smoking status, daily protein intake, number of chronic diseases, serum total cholesterol level, and plasma glucose level, and mean blood pressure), except for the variable used in each subgroup analysis.

## Discussion

The prevention of frailty is an important strategy through which to address related comorbidities, such as falls, cardiovascular events, cognitive impairment, and mortality [38, 39]. The cycle of frailty consists of four main components: reduced resting metabolic rate, decreased total energy expenditure, chronic undernutrition, and sarcopenia [40]. Of these, sarcopenia is of particular importance to enabling activities of daily living, preventing falls, and reducing various metabolic diseases [39, 40]. Therefore, methods for the early detection and prevention of sarcopenia would be of clinical and societal value.

In this study, adults of both sexes who had higher TyG index values were more likely to have LSMI, even after adjusting for age and other confounding factors. These findings were based on data from a representative, nationwide, cross-sectional survey. The exact mechanism by which TyG index is positively associated with LSMI is not known, although we hypothesize that insulin resistance and chronic inflammation may be the major links between elevated TyG index and an increased risk of sarcopenia. The pathogenesis of sarcopenia has been suggested to be closely related to chronic inflammation [20], which consequently increases insulin resistance [41], and may be reflected as an increased TyG index. The TyG index is thought to represent insulin resistance because it is calculated on the basis of two metabolic parameters: serum triglycerides and fasting glucose. Although we could not directly investigate the association between TyG index and insulin resistance index due to a lack of insulin data in the 2008–2011 KNHANES, we did find that the TyG index was related to the severity of metabolic syndrome, which is closely associated with insulin resistance [24, 42]. In addition, we found that a higher TyG index was associated with higher blood leukocyte count, a marker of chronic inflammation [43].

Sarcopenia is accompanied by muscle fat accumulation and an increase in pro-inflammatory cytokines, such as interleukin-6 (IL-6) and tissue necrosis factor alpha (TNF-α) within myocytes [44, 45], which contribute to subsequent decreases in muscle mass and strength [46, 47]. IL-6 downregulates glucose transporter 4 expression and insulin receptor substrate-1 (IRS-1), resulting in reduced transport of glucose into cells (including myocytes) and aggravation of insulin resistance [48]. TNF-α initiates a wide range of downstream signaling cascades, such as the activation of nuclear factor kappa B (NF-κB) and c-Jun N-terminal kinase (JNK) [49, 50]. In turn, NF-κB and JNK lead to the impairment of IRS-1 and aggravation of insulin resistance [49]. Moreover, upregulated NF-κB caused by pro-inflammatory cascades causes ubiquitination of muscle proteins and dissociation of actin and myosin filaments, which consequently leads to further loss of skeletal muscle [51, 52].

In subgroup analysis, the TyG index was independently related to LSMI regardless of the presence of several risk factors for sarcopenia, such as sex, smoking status, and protein intake status [53–55]. When we divided study subjects according to age, adults under 65 years had higher odds of LSMI with increasing higher TyG index tertile; however, there was no relationship between TyG index and LSMI in adults over 65 years of age. Although we could not explain the exact cause, the different percentage of participants with at least one chronic disease could account for the difference between younger and old adults (4.2% in younger adults group vs. 21.3% in older adult group). Regular exercise has positive effects on muscle mass and decrease insulin resistance and chronic inflammation [56, 57]. In our study, the regular exercise group showed a weakened relationship between TyG and LSMI (overall *P* = 0.087), compared to the non-regular exercise group. However, the ORs for LSMI showed consistent linearity with increases in TyG index tertile in both groups (*P* for trend = 0.027, < 0.001). Because the IPAQ does not provide information on the timing of exercise and or type/duration of physical activity, further research is needed with more detailed information on physical activity.

The relationship between the TyG index and LSMI was only significant in the normal alcohol consumption group (< 30 g/day). Prior evidence has suggested that heavy alcohol drinking may accelerate sarcopenia [58], and it may interfere with the relationship between TyG index and LSMI in the excessive consumption group (≥30 g/day). When we divided the study group according to BMI, only the normal weight group showed a significant relationship between TyG index tertile and LSMI. Obese people are at risk for being exposed to oxidative stress and chronic inflammation [59], and this chronic inflammatory status could contribute to TyG index values in various ways, such as glucose uptake and adipokine secretion [60, 61]. The number of individuals in the underweight group was small, and small sample size could act as confounding factor in the statistical analysis. Further research with a larger number of study participants would be helpful to elucidating the relationship between TyG index and LSMI in underweight individuals.

Unlike aging, alcohol drinking, exercise, underweight, and obesity are modifiable risk factors. Therefore, reducing alcohol consumption, regular exercising, and maintaining normal weight could be priority strategies to preventing LSMI.

### Strengths and limitations

This is the first study to confirm a relationship between the TyG index and muscle mass through the use of DXA and a representative, nationwide dataset from Korean adults. However, this study had several limitations. First, data on muscle mass information was only collected; this study could not obtain muscle strength or performance data, precluding a direct diagnosis of sarcopenia among the adults. Second, the study could not compare TyG index values with insulin resistance index values, such as homeostatic model assessment for insulin resistance. Finally, due to the cross-sectional study design, we could not assess causality between TyG index and LSMI.

## Conclusion

In conclusion, this study found that the TyG index is independently and negatively associated with LSMI in Korean adults over 19 years old. Because the TyG index can be easily measured in clinical settings through blood sampling, this may serve as a helpful method for the early detection of sarcopenia and related comorbidities, thus enabling timely initiation of treatment and reduced social, healthcare expenditures for treating sarcopenia. Longitudinal cohort studies and exploratory studies are needed to investigate the relationship between the TyG index and sarcopenia.

## Supplementary Information

**Additional file 1. Table S1.** Clinical characteristics of the study population without dual energy X-ray absorptiometry data. **Table S2.** Clinical characteristics of the study population with or without dual energy X-ray absorptiometry data.

## Data Availability

The dataset used in this study (KNHANES) can be obtained from the Korea Centers for Disease Control and Prevention (http://www.cdc.go.kr/CDC/eng/main.jsp) after submission and evaluation of an appropriate research proposal.

## Abbreviations

TyG: Triglyceride-glucose
LSMI: Low skeletal muscle mass index
KNHANES: Korea National Health and Nutrition Examination Survey
DXA: Dual-energy X-ray absorptiometry
HDL: High-density lipoprotein
WBC: White blood cell
ASM: Appendicular skeletal muscle mass
SMI: Skeletal muscle mass index
BMI: Body mass index
FNIH: Foundation for the National Institutes of Health
IPAQ: International Physical Activity Questionnaire
FFQ: Food frequency questionnaire
CHO: Carbohydrate
SE: Standard error
ORs: Odds ratios
Cis: Confidence intervals
IL-6: Interleukin-6
TNF- α: Tissue necrosis factor alpha
IRS-1: Insulin receptor substrate-1
NF-κB: Nuclear factor kappa B
JNK: c-Jun N-terminal kinase
