# Supplementary material for: Inverse association between triglyceride glucose index and muscle mass in Korean adults: 2008–2011 KNHANES
Source: Lipids Health Dis. 2020 Nov 22;19:243. doi: 10.1186/s12944-020-01414-4 (PMC7682063; doi:10.1186/s12944-020-01414-4)
Supplement: Supplementary file 1 — Additional file 1. Table S1. Clinical characteristics of the study population without dual energy X-ray absorptiometry data. Table S2. Clinical characteristics of the study population with or without dual energy X-ray absorptiometry data. [file 12944_2020_1414_MOESM1_ESM.docx]

**Supplementary table 1. Clinical characteristics of the study population without dual energy X-ray absorptiometry data**

|  | **2008–2011 KNHANES** | | | | |
| --- | --- | --- | --- | --- | --- |
| **TyG index** | **T1**  (6.57–8.18) | **T2**  (8.19–8.71) | **T3**  (8.72–11.78) | **Total** | *P* for trend |
| **N** | 2127 | 2074 | 2168 | 6369 |  |
| **Male sex, % (SE)** | 34.6 (1.4) | 50.9 (1.5) | 62.2 (1.3) | 48.9 (0.8) | <0.001 |
| **Age, years** | 39.3 ± 0.4 | 44.8 ± 0.5 | 47.9 ± 0.5 | 44.0 ± 0.4 | <0.001 |
| **Waist circumference, cm** | 75.9 ± 0.3 | 80.9 ± 0.3 | 86.2 ± 0.3 | 81.0 ± 0.2 | <0.001 |
| **Mean blood pressure, mmHg** | 85.3 ± 0.3 | 89.5 ± 0.4 | 93.8 ± 0.3 | 89.6 ± 0.2 | <0.001 |
| **Leukocyte count (*1,000/μL)** | 5.7 ± 0.1 | 6.2 ± 0.1 | 6.7 ± 0.1 | 6.2 ± 0.0 | <0.001 |
| **Glucose, mg/dL** | 87.4 ± 0.2 | 91.7 ± 0.2 | 96.5 ± 0.3 | 91.9 ± 0.2 | <0.001 |
| **Log-transformed Triglyceride, mg/dL** | 4.0 ± 0.0 | 4.6 ± 0.0 | 5.3 ± 0.0 | 4.7 ± 0.0 | <0.001 |
| **Employment status, % (SE)** | 60.9 (1.5) | 62.8 (1.5) | 66.5 (1.4) | 63.4 (1.0) | 0.010 |
| **Heavy alcohol use, % (SE)** | 5.2 (0.7) | 8.4 (0.8) | 13.8 (1.0) | 9.1 (0.5) | <0.001 |
| **Current smoker, % (SE)** | 18.6 (1.3) | 28.6 (1.6) | 37.9 (1.5) | 28.0 (0.8) | <0.001 |
| **Regular exercise, % (SE)** | 24.8 (1.3) | 21.4 (1.3) | 20.0 (1.2) | 22.1 (0.8) | 0.015 |
| **Daily calorie intake, kcal/day** | 1937.7 ± 27.9 | 1990.1 ± 29.0 | 2120.6 ± 32.8 | 2016.2 ± 20.0 | <0.001 |
| **Daily protein intake, % of total calorie intake** | 14.6 ± 0.1 | 14.4 ± 0.2 | 14.2 ± 0.1 | 14.4 ± 0.1 | 0.092 |
| **SMI** |  |  |  |  | N/A |
| **Men** | - | - | - | - |  |
| **Women** | - | - | - | - |  |
| **Number of components of metabolic syndrome** |  |  |  |  | <0.001 |
| **0** | 58.4 (1.3) | 33.6 (1.4) | 3.3 (0.5) | 32.2 (0.8) |  |
| **1** | 30.0 (1.2) | 36.5 (1.4) | 14.2 (1.0) | 26.8 (0.7) |  |
| **2** | 9.0 (0.7) | 20.5 (1.2) | 32.2 (1.5) | 20.4 (0.6) |  |
| **3** | 2.2 (0.3) | 7.1 (0.7) | 29.0 (1.4) | 12.7 (0.5) |  |
| **4** | 0.3 (0.1) | 2.1 (0.4) | 16.6 (1.0) | 6.3 (0.4) |  |
| **5** | - | - | 4.8 (0.6) | 1.6 (0.2) |  |
| **Number of chronic diseases, % (SE)** |  |  |  |  | 0.058 |
| **0** | 93.2 (0.8) | 90.8 (0.8) | 90.2 (0.7) | 91.3 (0.5) |  |
| **1** | 5.9 (0.7) | 8.4 (0.7) | 8.7 (0.7) | 7.7 (0.4) |  |
| **≥2** | 0.9 (0.3) | 0.8 (0.2) | 1.1 (0.3) | 0.9 (0.2) |  |

Abbreviations: TyG index, triglyceride-glucose index; KNHANES, Korean National Health and Nutrition Examination Survey; SE, standard error; BMI, body mass index; SMI, skeletal muscle mass index; N/A, not applicable.

*P* for trend was derived from weighted generalized linear regression analysis for continuous variables and weighted chi-square test for linear-by-linear association for categorical variables.

**Supplementary table 2. Clinical characteristics of the study population with or without dual energy X-ray absorptiometry data**

|  | **2008–2011 KNHANES** | | | |
| --- | --- | --- | --- | --- |
|  | **with DXA** | **without DXA** | **Total** | *P* for trend |
| **N** | 15741 | 6369 |  |  |
| **Male sex, % (SE)** | 49.3 (0.5) | 48.9 (0.8) | 49.2 (0.4) | 0.703 |
| **Age, years** | 43.2 ± 0.3 | 43.9 ± 0.4 | 43.6 ± 0.2 | 0.186 |
| **Waist circumference, cm** | 80.2 ± 0.1 | 80.9 ± 0.2 | 80.6 ± 0.1 | 0.013 |
| **Mean blood pressure, mmHg** | 90.0 ± 0.2 | 89.5 ± 0.2 | 89.8 ± 0.2 | 0.066 |
| **Leukocyte count (*1,000/μL)** | 6.1 ± 0.0 | 6.2 ± 0.0 | 6.1 ± 0.0 | 0.002 |
| **Glucose, mg/dL** | 92.2 ± 0.1 | 91.8 ± 0.2 | 92.0 ± 0.1 | 0.076 |
| **Log-transformed Triglyceride, mg/dL** | 4.6 ± 0.0 | 4.6 ± 0.0 | 4.6 ± 0.1 | 0.901 |
| **Employment status, % (SE)** | 64.8 (0.6) | 63.4 (1.0) | 64.4 (0.5) | 0.224 |
| **Heavy alcohol use, % (SE)** | 8.9 (0.3) | 9.1 (0.5) | 8.9 (0.3) | 0.689 |
| **Current smoker, % (SE)** | 27.1 (0.6) | 28.0 (0.9) | 27.4 (0.5) | 0.403 |
| **Regular exercise, % (SE)** | 24.9 (0.5) | 22.1 (0.8) | 24.1 (0.5) | 0.006 |
| **Daily calorie intake, kcal/day** | 2040.1 ± 11.4 | 2015.2 ± 20.2 | 2027.6 ± 11.5 | 0.288 |
| **Daily protein intake, % of total calorie intake** | 14.4 ± 0.1 | 14.4 ± 0.1 | 14.4 ± 0.0 | 0.907 |
| **SMI** |  |  |  | N/A |
| **Men** | 0.9558 ± 0.0024 | - | 0.9558 ± 0.0024 |  |
| **Women** | 0.6381 ± 0.0017 | - | 0.6381 ± 0.0017 |  |
| **Number of components of metabolic syndrome** |  |  |  | 0.100 |
| **0** | 30.8 (0.5) | 32.2 (0.8) | 31.2 (0.5) |  |
| **1** | 29.3 (0.5) | 26.8 (0.7) | 28.6 (0.4) |  |
| **2** | 19.2 (0.4) | 20.4 (0.6) | 19.6 (0.3) |  |
| **3** | 12.8 (0.4) | 12.7 (0.5) | 12.8 (0.3) |  |
| **4** | 6.3 (0.2) | 6.3 (0.4) | 6.3 (0.2) |  |
| **5** | 1.6 (0.1) | 1.6 (0.2) | 1.6 (0.1) |  |
| **Number of chronic diseases, % (SE)** |  |  |  | <0.001 |
| **0** | 93.5 (0.3) | 91.3 (0.5) | 92.9 (0.2) |  |
| **1** | 5.9 (0.2) | 7.7 (0.4) | 6.4 (0.2) |  |
| **≥2** | 0.6 (0.1) | 0.9 (0.2) | 0.7 (0.1) |  |

Abbreviations: TyG index, triglyceride-glucose index; KNHANES, Korean National Health and Nutrition Examination Survey; SE, standard error; BMI, body mass index; SMI, skeletal muscle mass index; N/A, not applicable. *P* for trend was derived from weighted generalized linear regression analysis for continuous variables and weighted chi-square test for linear-by-linear association for categorical variables.
